# Differentiated thyroid carcinoma – “a blessing or a curse”

**DOI:** 10.1186/1756-6614-8-S1-A1

**Published:** 2015-06-22

**Authors:** Zbigniew Adamczewski, Andrzej Lewiński

**Affiliations:** 1 Department of Endocrinology and Metabolic Diseases, Polish Mother’s Memorial Hospital – Research Institute, Lodz, Poland

## 

Among the different thyroid carcinomas, papillary carcinoma (PTC) is the most common malignant tumor of the thyroid gland, as well as the most common malignancy among all cancers of the endocrine glands. It is a cancer which - among other cancers - is characterized by clearly defined rules of diagnosis, treatment and monitoring (follow-up). One of the most important features of PTC is its slow growth and spread (PTC metastasizes very late, very slowly). The result is that in the majority of cases, cancer foci diagnosed exclusively in the thyroid gland (pT1aN0M0) are subjected to surgical treatment. Treatment is based on the performance of total thyroidectomy and - next - radioiodine ^131^I use, the latter to destroy the thyroid remnants - the source of thyroglobulin - for "clearing the foreground". After completion of therapy, thyroglobulin in these patients is becoming a tumor marker which is used to assess the effectiveness of therapy. The evaluation of thyroglobulin concentration after prior stimulation with TSH is the most sensitive method speaking for or against the presence of thyroid tissue in the possible metastases.

Patients with differentiated thyroid carcinoma (especially with papillary cancer) are usually informed at the time of diagnosis that such a diagnosis is “a blessing in disguise”. That means that the risk of dying from their disease is lower than in the case of communication accidents, and that they should not be afraid of this disease, just as usually they are not afraid of normal everyday activities like “leaving home”. In other words, that means that in their case, just this type of cancer and not another, is a real blessing. Unfortunately, a very good prognosis, resulting from the aforementioned slow cancer growth, even then carries the risk of recurrence of malignancy, and the recurrence time can be as long as the patient's life is. These patients live all the time with the stigma of the cancer, the symptoms of which we will be looking for in their bodies - as doctors, despite the passing years. It can be treated, in a sense, as “the curse of cancer”. Each patient’s next meeting with his/her doctor, whether in an out-patient clinic or in a hospital ward, for the patient is the reason for subsequent stress. It brings to mind all the worst, associated with the words "you suffer from cancer", words spoken once for the first time a long time ago. One should not forget about all of this and need to keep empathy, seeking to make such diagnostic methods, which are the least onerous for the body of the patient, methods which at the same time will allow for a quick and reliable assessment of disease activity (time to wait for the result drags on exceptionally), empowering the patient to the conclusion that “I am healthy! ... until the next test ...”.

Currently the preferred diagnostic method in monitoring differentiated thyroid cancer is to assess thyroglobulin concentration after TSH stimulation. One way to achieve a high TSH level is to withdraw L-T4, i.e. induction of endogenous thyrotropinemia (putting the patient in a state of primary hypothyroidism). However, the symptoms of hypothyroidism, which are highly unpleasant for the patient and fuel a depressive attitude, appearing during almost complete L-T4 deficiency, are often interpreted by patients as a manifestation of cancer relapse. The period of preparation for tests, often lasting for more than a month is an endless band of memories and reflections on the incurable disease. In this connection, doctor should seek to determine thyroglobulin concentration in the condition of exogenous stimulation with recombinant human TSH (rhTSH). This way of preparing for the test is safe and reliable for the patient, devoid of any adverse circumstances related to the stimulation with endogenous TSH. By that means, patients can reduce the burden related to disease consciousness and they more frequently mention the “blessing” than the “curse” associated with the established earlier diagnosis of differentiated thyroid cancer. In addition, you need to be aware that the use of rhTSH is generally considered to be more effective in achieving optimum of iodine uptake by thyroid tissue, than the introduction of endogenous thyrotropinemia [1].

A separate issue is the problem, whether beingn forms of papillary tumors, not provided for in the existing classification of thyroid tumors [2], actually exist. Some authors are inclined to such a concept but the view prevails that these are various papillary variants of follicular adenomas [3], and not benign papillary neoplasms.
